# Systems Vaccinology in HIV Vaccine Development

**DOI:** 10.3390/vaccines10101624

**Published:** 2022-09-28

**Authors:** Jielin Zhang, Philip Askenase, Clyde S. Crumpacker

**Affiliations:** Allergy & Clinical Immunology, Yale School of Medicine, New Haven, CT 06510, USA; Department of Medicine, Beth Israel Deaconess Medical Center, Harvard Medical School, Boston, MA 02215, USA

## 1. Background

Themes of discussions in the Special Issue of T Cell Immunity and HIV-1 Pathogenicity are outlined here. We hope the contributions of investigators in multidisciplinary fields will become a milestone for advancing vaccinology. This could lead to improved immunity at the cellular level that will constitute the functional units, resources, and powerhouse of successful vaccination.

Vaccinology was established before immunology and virology [1,2,3]. For historical reasons, the production of vaccines has heavily depended on empirical and serendipitous processes until the last decade, in which computer technology shifted the basis of how scientific information is obtained, exchanged, and made available, including data processing, knowledge extraction, conceptualization, and theorization [4,5,6,7,8].

Vaccines remain one of the most successful medical advances in modern times, and vaccination has transformed public health. Vaccines can elicit the highly evolved, extraordinary ability of the human immune system to respond to, remember, and counteract invading pathogens, such as viruses, bacteria, and parasites, etc. Vaccination can prevent diseases and their complications.

Vaccination relies on a nexus response between an invader and our body, (1) to utilize the invader in the format of a vaccine to prime our intrinsic immune system to remember the intruder/pathogen/antigen and (2) to launch a local or systemic immune protection in time to prevent the invader from damaging our body.

Four decades of HIV/AIDS research has provided a great deal of knowledge on the immune system response to a naturally occurring viral infection, which infects the CD4 cells central to immune protection. Conversely, if we can develop new vaccines that reprogram a host anti-HIV response by restoring CD4 T cell function, this could lead to a cure for HIV.

In this Special Issue, we aim to have open discussions on (1) how to harness the success of coronavirus disease (COVID) mRNA vaccines in the development of a next generation of HIV/AIDS vaccines, (2) why a systemic vaccinology approach is needed to develop effective HIV/AIDS vaccines, and (3) the strategy of developing HIV/AIDS vaccines via the approach of systems vaccinology, which has a profound impact in today’s personalized/precision medicine. We outline the themes enumerated above as follows to facilitate the discussion.

## 2. COVID mRNA Vaccines and Systems Vaccinology

The strength of systems vaccinology is at the vaccine design level, built on multidisciplinary mega-data analyses to delineate the immune signaling pathways initiated by a pathogen up to the immune responses that clear off or control the pathogen replication. The approach of systems vaccinology can also be used to delineate the immune responses after vaccination. The latter is applied to the study of immune responses elicited by COVID-19 mRNA vaccines. One study shows a robust production of neutralizing antibodies and significant increases in antigen-specific polyfunctional CD4 and CD8 T cells after the second dose of vaccination by Pfizer-BioNTech mRNA vaccine (BNT162b2) [9].

Further basic studies from bench, animal models, and clinical trials can deepen the data of systems vaccinology. This will elucidate the pathogenicity of SARS-CoV-2, provide insights on long COVID-19 and explore pathways where vaccine-elicited treatments of COVID-19, antiviral drugs, and biologics can combine to improve treatment outcomes [10,11,12,13].

## 3. Developing HIV Vaccine via Systems Vaccinology

A myriad of data from more than 4 decades of HIV/AIDS research on HIV pathogenicity and T cell biology, derived from Center for AIDS Research (CFAR) investigators, AIDS clinical trials group (ACTG) labs, and AIDS vaccine clinical trials, are the high-input data for the systems vaccinology approach to vaccine development. This will not only aid the rational design of AIDS vaccines but also greatly increase the vaccine efficacy, especially that of vaccine designs based on the gain-of-function model that exemplify reprogramming of host CD4 T-cell immunity elicited by a vaccine and embody anti-HIV function.

The strength of the systems vaccinology approach depends on its multidisciplinary mega-data, epitomized by the high-throughput, single-cell ‘omics’ technologies, specifically the data of transcriptomics/epigenomics, metabolomics, and mass cytometry, coupled with computational approaches to construct a global map of the complex processes that occur during an immune response to vaccination [14,15,16].

## 4. The Impact of Development of AIDS Vaccines via Systems Vaccinology in Modern Medicine

Unlike the development of COVID-19 mRNA vaccines, the next generation of AIDS vaccines will not test its efficacy by human clinical trials, but will be achieved by understanding its efficacy via meeting the pillar indexes of successful vaccines by systems vaccinology approach first, and then examining the vaccine-induced gain-of-function in host immunity, by HIV-specific CD4 T cell clonal formation and expansion [17]. Such strategies let the host CD4 T cells do what they do best, i.e., control the viral pandemic as they have achieved with COVID-19 vaccination.

Specifically, we suggest integrating pillar indexes of vaccine efficiency in a systems vaccinology approach for developing HIV/AIDS vaccines. They are a mechanistic immunological correlate of protection (mCoP), non-mechanistic immunological correlate of protection (nCoP), correlate of risk (CoR), and surrogate of protection (SoP) [18,19,20,21]. These indexes also distinguish the hypersensitivity responses that occur in HIV infection, dubbed immune inflammation/exhaustion, and are related to the allergic, anergic, side effect, and vaccine safety or efficacy in vaccination.

In 2010, Bill Gates pledged $10 billion for vaccines over the next decade and said that he hoped the coming 10 years would be the decade of vaccines [22]. Systems vaccinology is rapidly developing with the advance in computer science [4,5,6,7,8]. For the first time, we have begun to understand the mechanisms by which highly successful vaccines mediate protective immunity, and we have also begun to harness such insights in designing new vaccines against global pandemics [23].

With the myriad of data in HIV/AIDS research from a loss-of-function model in human immunity, we can input data to the systems vaccinology approach and perform high-throughput gain-of-function analyses in order to achieve the output of effective vaccines for HIV/AIDS immunization. We recall and contrast the admonishment of Sydney Brenner for data analysis made decades ago: “We are drowning in a sea of data and thirsting for knowledge. Most biology today is low input, high throughput, no output biology” [23].

## 5. Conclusions

We can all pitch in to develop HIV/AIDS vaccines via the systems vaccinology approach. This will set up a model for the study of functions of other systems, specifically the human nervous system. The new knowledge and techniques will enable us to make significant impacts on personalized/precision medicine and to win humanity’s war against disease, related disability, and death. It is not only ethical to do this but also our duty.

## Data Availability

Not applicable.
